# Envy and Jealousy of Living-Apart-Together Relationships in Continuing Care Retirement Communities

**DOI:** 10.1093/geroni/igaa057.1231

**Published:** 2020-12-16

**Authors:** Chaya Koren, Liat Ayalon

**Affiliations:** 1 University of Haifa, Haifa, Israel; 2 Bar-Ilan University, Ramay Gan, HaMerkaz, Israel

## Abstract

Moving to a continuing care retirement community (CCRC) and living apart together (LAT) as a repartnering form, represent new late-life beginnings. A larger qualitative study on LAT relationships constructed in the CCRC identified envy and jealousy yet they were not examined in-depth. Envy is wanting something we lack whereas jealousy is fear of losing something that is ours to another. These emotions are rarely explored in the context of older adults’ relationships. Our aim is to examine experiences of envy and jealousy from perspectives of residents aged 79 to 96 and staff, heuristically using Goffman’s framework on (semi)-totalitarian institutions. 30 semi structured qualitative interviews were conducted in three CCRCs in Israel with 10 LAT residents, 10 residents not LAT, and 10 CCRC staff members including social workers. Analysis was conducted based on principles of thematic analysis and triangulation. Findings refer to kinds of envy, ignoring envy, and the development and consequences of jealousy and/or envy related to LAT in the CCRC. Conclusions address how semi-totalitarian CCRC features influence envy and jealousy experiences including implications for assisting social workers, older adults and their family members to adjust to life in the CCRC and assist CCRC management and staff to address possible consequences of envy and jealousy.

